# Gastric stump cancer after gastrectomy by gastroduodenal peptic ulcer

**DOI:** 10.1590/0102-6720201600010017

**Published:** 2016

**Authors:** Augusto DIOGO, Lucas Ferreira BOTELHO, Andréa NISHIYAMA, Letícia Eugênia ZUMPANO, Rosana Caldeira MONTE, Samantha Cunha ROSA

**Affiliations:** Digestive Surgery Service, Clinics Hospital, Federal University of Uberlândia Uberlândia, MG, Brazil

## INTRODUCTION

Gastric stump cancer was first reported as a disease entity by Balfour in 1922^1^^,^ 6. It was defined initially as cancer that arises in the remnant stomach after five years of gastrectomy for benign disease such as peptic ulcer^1^^,^ 6 ^,^ 8. Another opinion includes ten-year latency period after primary operation for benign or malignant disease. Cancer recurrence in the stump after ten years of gastrectomy is rare^9^.

The anatomical region most commonly affected is next to the anastomosis, gastric side. With a prevalence of 1-9% among cases of stomach cancer, it affects between 0.8-8.9% of patients who underwent partial gastric resection for peptic ulcer^1^.

Reduction in the prevalence of peptic ulcer gastrectomy is reported^4^^,^ 8 ^,^ 10 due to therapeutic advances, among them drug treatment by *Helicobacter pylori* infection^8^^,^ 10. However, surgery continues to be performed for the complications of peptic ulcer disease, such as perforation, bleeding or stenosis^8^.

In this article, the authors report two cases of gastric stump cancer after Billroth II gastrectomy for peptic ulcer disease.

## CASE REPORTS

### Case 1

**FIGURE 1:**
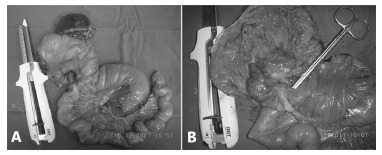
- Product of gastrojejunal resection: A) before the opening of the surgical specimen, and B) with mucosal exposure

Man, 58, was admitted with hemodynamic instability episodes due to hematemesis and melena, about two weeks. Endoscopy revealed ulcerative-vegetative lesion with elevated borders and infiltrated in gastric stump, with a history of partial gastrectomy Billroth II for more than 20 years for bleeding peptic ulcer. Biopsy revealed invasive gastric adenocarcinoma. He underwent total gastrectomy for gastric remnant (Figure 1) and reconstruction by esophagojejunal terminolateral anastomosis with circular stapler and jejunojejunal laterolateral with linear stapler, and lymphadenectomy, splenectomy and hepatectomy wedge, as a result of tumor infiltration in the left hepatic lobe. Patient died after 10 months of postoperative follow-up.

### Case 2

Man, 71, underwent partial gastrectomy Billroth II for more than 30 years due to gastric ulcer. It was admitted complaining bloating, vomiting, epigastric pain and weight loss of 5 kg in two months. Endoscopy showed infiltrative lesion in anastomotic edge and biopsy showed moderately differentiated adenocarcinoma. Underwent total gastrectomy, lymphadenectomy to D2, splenectomy, omentectomy and enterectomy in block. The reconstruction of the digestive tract was done through esophagojejunal terminolateral anastomosis with circular stapler, and enteroanastomosis jejunojejunal 5 cm from the duodenojejunal angle done manually. On the 1^st^ day of postoperative period progressed with hemodynamic instability and was referred to the intensive care unit. Underwent endoscopy that showed presence of signs suggestive of ischemia in esophagojejunal anastomosis. It was decided to keep him in conservative medical treatment and total parenteral nutrition, but died on the 30^th^ day after surgery.

## DISCUSSION

Partial gastrectomy for peptic ulcer disease is a risk factor for gastric cancer^8^. The main pathogenesis of gastric stump cancer is biliary-pancreatic reflux causing chronic inflammation of the remaining mucosa, developing into atrophic gastritis, intestinal metaplasia and displasia^1^. Other possible causes are: 1) hypo- or achlorhydria, resulting in bacterial overgrowth and increased exposure of carcinogenic agents, for example, nitrosamines^7^^,^ 8; 2) effect of hormonal regulation after vagotomy and hipogastrinemia^7^; 3) presence of surgical suture^1^; and 4) Epstein-Barr virus. The latter occurs most commonly in the gastric stump, unlike *Helicobacter pylori* infection, more frequent in the intact stomach^8^.

The *H. pylori* infection is considered the main risk factor for adenocarcinoma^2^. However, its role in gastric stump cancer is unlikely, since the infection rate in these patients is lower. Furthermore, the gastric remnant is unfavorable environment for the colonization of microorganisms because of biliopancreatic alcaline reflux^5^^,^ 6.

There is a larger number of cases of gastric stump cancer after reconstruction Billroth II, when compared to Billroth I. This is explained by the fact that in the first place inflammation and regeneration of the gastric mucosa, the persistent contact of the anastomosis of gastric stump with the biliary acids^9^.

The most common location of the tumor in the remnant stomach depends on the type of operation carried out previously. When the technique is Billroth II, it is more frequent in the anastomosis area, while in Billroth I in non anastomotic^9^.

The interval between initial gastrectomy and diagnosis of gastric cancer stump is greater when the first operation was due to benign disease, than to malignant^6^^,^ 9 ^,^ 10. This observation is probably a result of the difference in age of the patient when the initial gastrectomy was done, in both groups, as peptic ulcer usually occurs in younger patients than cancer^6^^,^ 10.

Survey conducted in 95 institutions in Japan found more cases between 10-15 years of operation in patients whose initial lesion was malignant. In gastrectomy for peptic ulcer disease the peak incidence occurred around the fourth decade after operation^9^.

Gastric stump cancer surgically treated has bad prognosis. Was observed lower five-year survival in patients with gastric stump cancer than those with primary gastric cancer^10^. The treatment of choice is surgical D2 resection of remaining stomach, plus lymphadenectomy including organs and other adjacent lymph nodes resection^3^.

To improve results is necessary early diagnose. Therefore, endoscopic surveillance should be considered^10^. However, there is no consensus in the literature on the screening of gastric stump cancer after gastrectomy. For some, the endoscopic surveillance program should start one year till at least ten years^6^. For others, gastrectomy for peptic ulcer disease should continue beyond ten years^4^^,^ 6 ^,^ 10. But everyone agrees on the need for early cancer detection and appropriate follow-up program^10^.
